# Significant changes of the mechanical medial proximal tibial angle in dependence of internal and external rotation of the hinge axis in slope correcting infratuberositary tibial deflexion osteotomy

**DOI:** 10.1002/ksa.70219

**Published:** 2025-12-07

**Authors:** Lukas Jud, Malte Kölle, Georgios Neopoulos, Lazaros Vlachopoulos, Sandro F. Fucentese

**Affiliations:** ^1^ Department of Orthopedics Balgrist University Hospital, University of Zurich Zurich Switzerland

**Keywords:** anterior closing wedge, high tibial osteotomy, posterior tibial slope (PTS), slope correction

## Abstract

**Purpose:**

Tibial deflexion osteotomy (TDO) is performed to correct an increased posterior tibial slope (PTS). Unintended rotation of the osteotomy and the hinge axis (HA) orientation can result in a postoperative deviation of the mechanical medial proximal tibial angle (mMPTA). This study aimed to investigate how internal and external HA rotations affect postoperative mMPTA.

**Methods:**

Three‐dimensional (3D) bone models of ten patients with increased PTS were used to simulate infratuberositary TDO with different HA orientations and closing distances. Postoperative changes in mMPTA were analysed.

**Results:**

In total, 440 TDOs were simulated. The PTS changed by 0.9 ± 0.0° per mm of closing distance. TDO perpendicular to the coronal plane of the long‐leg radiograph showed no significant change in the postoperative mMPTA. Internal and external rotation of the HA resulted in significant changes in postoperative mMPTA, with absolute changes up to 4.5° ± 0.5°.

**Conclusion:**

A TDO oriented perpendicular to the leg's coronal plane preserves the preoperative mMPTA and therefore avoids unintended coronal correction. The mMPTA changed significantly with a rotation of the HA of only 5° and exceeded a postoperative change of ≥2° with 15° of HA rotation.

**Level of Evidence:**

N/A.

**Abbreviations**
2D: two‐dimensional
3D: three‐dimensional
ACL: anterior cruciate ligament
CT: computed tomography
HA: hinge axis
ICC: intraclass correlation coefficient
mMPTA: mechanical medial proximal tibial angle
PCL: posterior cruciate ligament
PSI: patient‐specific instruments
PTS: posterior tibial slope
TDO: tibial deflexion osteotomy

## INTRODUCTION

An increased posterior tibial slope (PTS) is a risk factor for graft failure after anterior cruciate ligament (ACL) reconstruction [13]. Tibial deflexion osteotomy (TDO) is considered for revision ACL reconstructions [3, 12], with improvements in patient reported outcomes and low graft rupture rates also reported [1, 4, 14, 23, 24]. Among other complications, changes in postoperative coronal alignment and mechanical medial proximal tibial angle (mMPTA) have been reported [2, 16]. The change in mMPTA can be explained by the different hinge axis (HA) orientations [25]. However, when performed conventionally under fluoroscopic guidance, achieving accurate HA orientation can be challenging. Typically, the tibial tuberosity and lateral knee radiographs are used to determine the orientation of the osteotomy plane [7, 18]. In such a technique, unintended changes in mMPTA due to rotational deviations in HA orientation may not completely be avoided under two‐dimensional (2D) fluoroscopy control. A three‐dimensional (3D) approach combined with intraoperative navigation may improve accuracy in such situations. This study quantified the effect of different HA orientations on postoperative mMPTA in TDO with various common osteotomy closing distances using a 3D computer simulation approach. We hypothesised that internal and external rotations of the HA would result in significant and relevant changes in postoperative mMPTA.

## MATERIALS AND METHODS

This study was approved by the local ethics committee (Zurich Cantonal Ethics Commission, BASEC‐NR. 2023‐00389), and all patients provided informed consent for participation in the study and its publication.

### Study design

**Figure 1:**
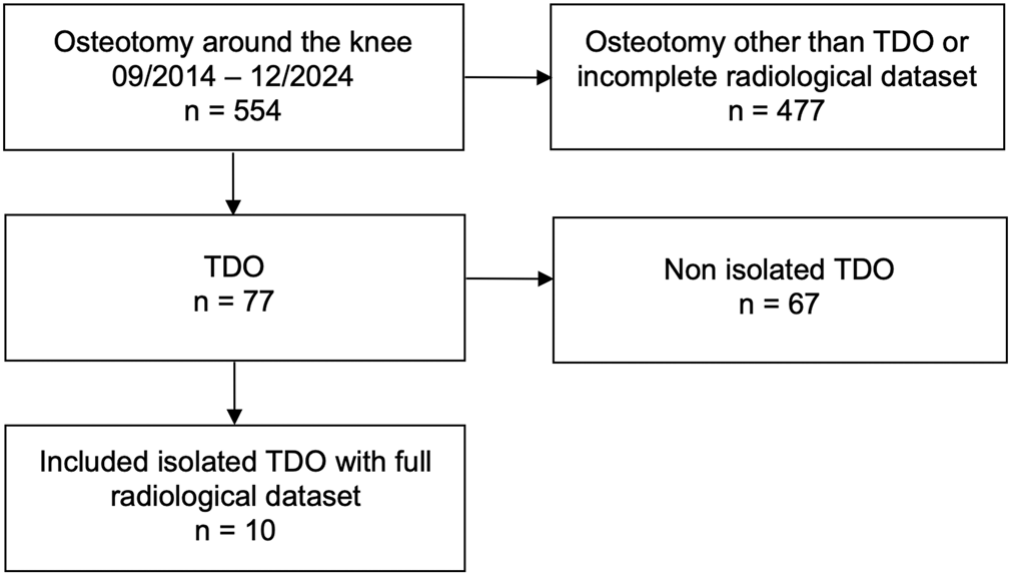
Flow chart of inclusion and exclusion of the patients. TDO, tibial deflexion osteotomy.

This was a retrospective study. Patients were selected from a database of patients who underwent osteotomy around the knee between September 2014 and December 2024 at our institution. Inclusion criteria consisted of an isolated TDO without concomitant coronal or rotational correction, a PTS ≥ 12°, available preoperative true lateral knee radiographs, and preoperative computed tomography (CT) scans of the lower extremity including the hip‐, knee‐ and the ankle‐joints. A flow chart of the inclusion and exclusion criteria is shown in Figure 1. After applying these criteria, ten knees (seven right and three left knees) from ten patients (seven males and three females) were included. The mean age was 27.7 ± 9.0 years (range: 18.1–47.2), and the mean body mass index (BMI) was 25.4 ± 5.5 kg/m² (range: 19.3–39.9). The preoperative mean PTS was 13.3 ± 1.8° (range: 12.0–18.1), whereas the preoperative mean mMPTA was 88.4 ± 1.2° (range: 86.5–90.1).

### Simulations and measurements

**Figure 2:**
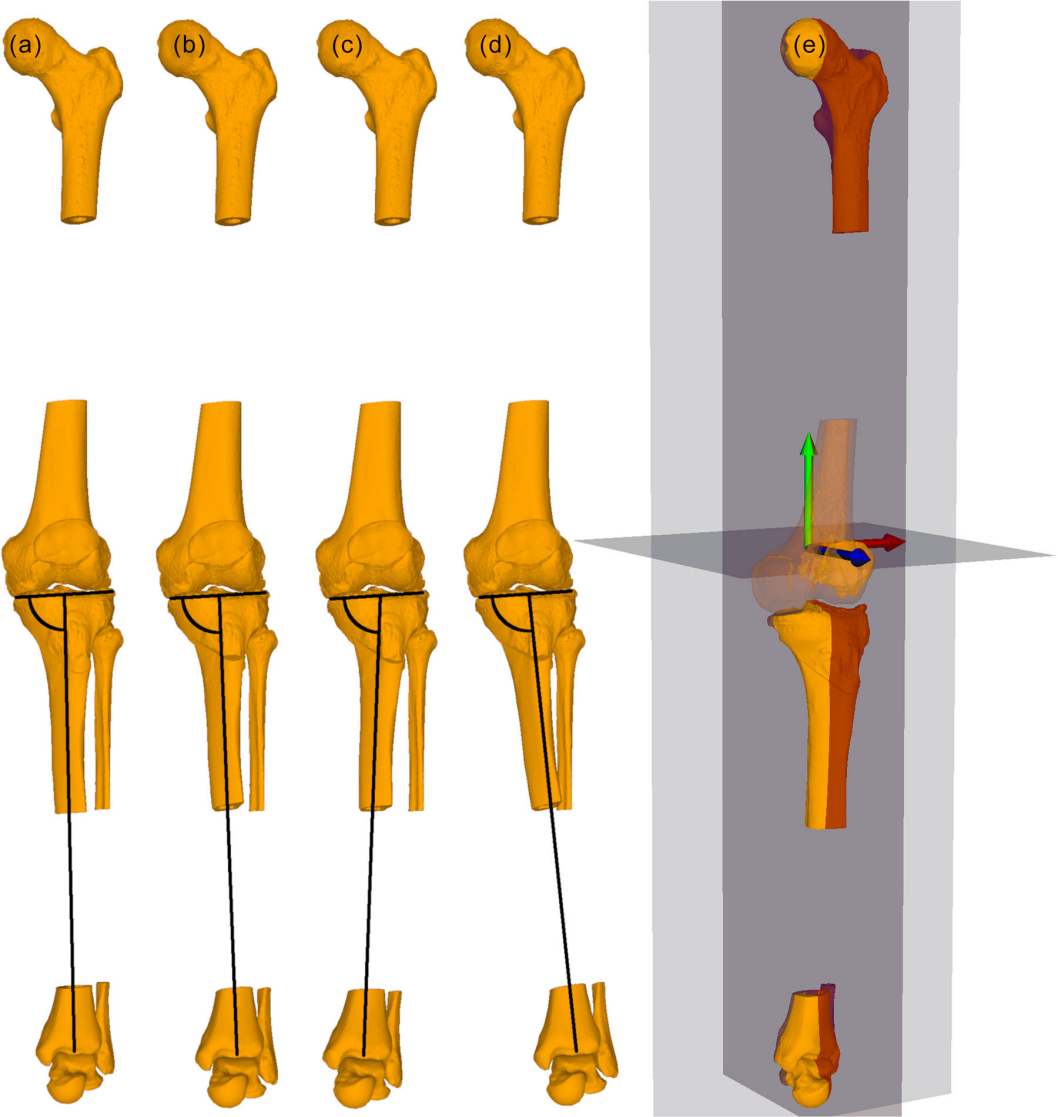
(a) Three‐dimensional (3D) bone model before the tibial deflexion osteotomy (TDO) (mechanical medial proximal tibial angle (mMPTA) measured 88.8° in this case). (b) Osteotomy performed using the baseline TDO with a closing distance of 12 mm (mMPTA is preserved at 88.8°). (c) TDO performed with an internal rotation of 30° and a closing distance of 12 mm (mMPTA measured 84.4°). (d) TDO performed with an external rotation of 30° and a closing distance of 12 mm (mMPTA measured 93.2°). The corresponding mMPTA values are schematically indicated in black. (e) The 3D model aligned using the reference coordinate system of the planning software. Reference planes (coronal, sagittal and axial) are shown in grey.

**Figure 3:**
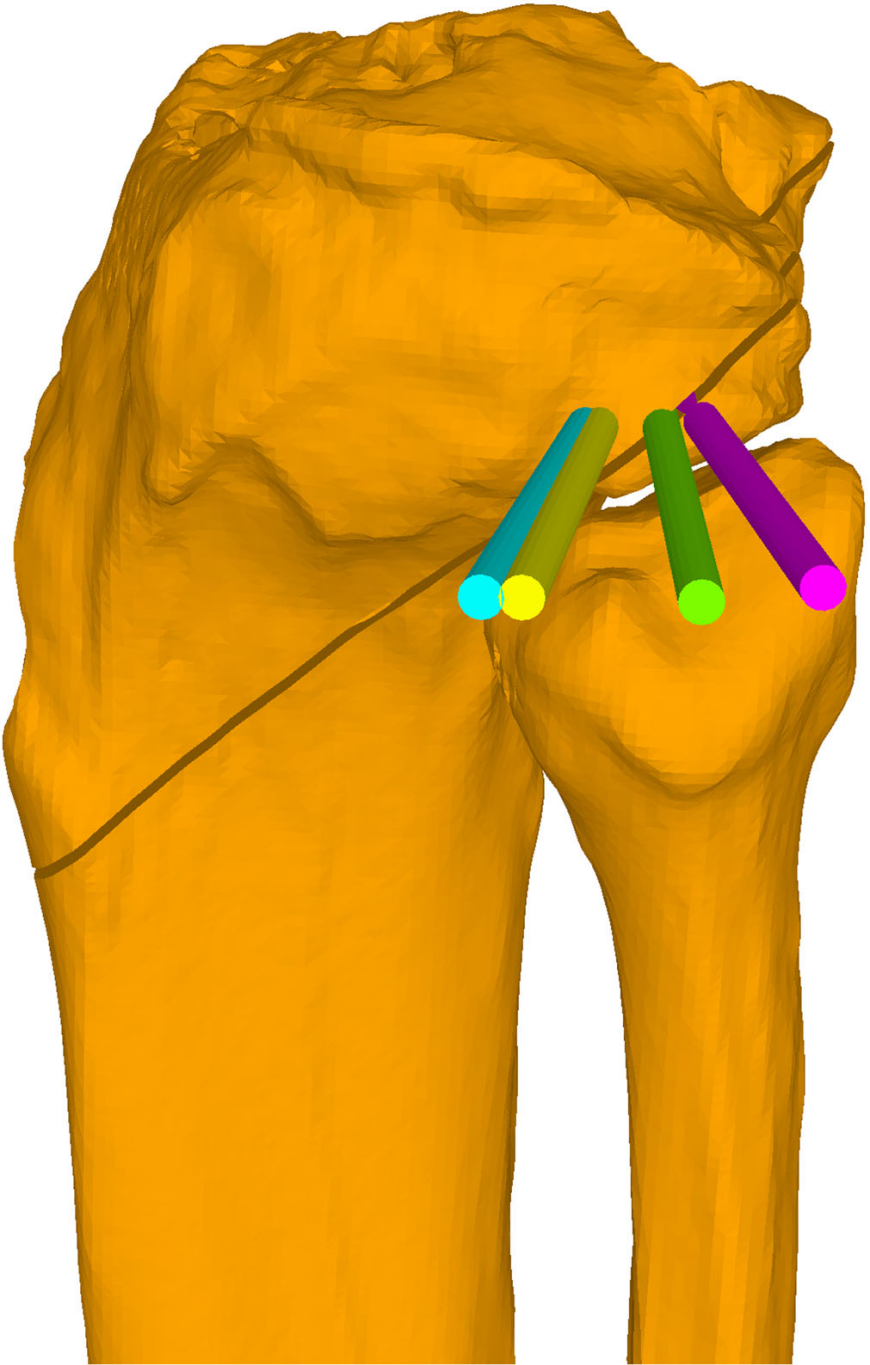
Different hinge axes (HA). The green line represents the HA of the baseline tibial deflexion osteotomy (TDO) (i.e., HA orientation that preserves the preoperative mechanical medial proximal tibial angle). The purple line represents the HA of a TDO perpendicular to the tibial tuberosity (i.e., externally rotated). The yellow line represents the HA of a TDO perpendicular to the lateral knee radiograph with a perfect overlay of the posterior tibial condyles (i.e., internally rotated). The turquoise line represents the HA of a TDO perpendicular to a lateral knee radiograph orientation with a perfect overlay of the posterior femoral condyles (i.e., internally rotated).

Preoperative PTS was measured on lateral knee radiographs as the angle between the medial tibial plateau and proximal anatomical axis of the tibia using two circles along the tibial diaphysis at 5 and 15 cm below the tibial plateau [12]. CT data of the lower extremity of the included patients were used to create 3D surface models of the lower extremities. The 3D bone models were imported into the in‐house surgical planning software CASPA (version 5.32 ×64, Balgrist CARD AG, Zurich, Switzerland). These models of the lower extremities were oriented with the reference coordinate system of CASPA, aligning the mechanical leg axis with the y‐axis and perpendicular to the axial plane. Next, the 3D models were rotated around the y‐axis until the anterior surface of the patella was aligned with the z‐axis (i.e., the patella facing directly forward), replicating the standardised long‐leg‐radiograph position. This method has been used and validated previously in several studies [6, 10, 11]. The HA for the baseline TDO was defined as a line perpendicular to the sagittal plane, positioned 10 mm anterior to the posterior cruciate ligament (PCL) insertion and 15 mm below the articular surface. The baseline TDO plane was defined as perpendicular to the HA, with the entry point immediately below the tibial tuberosity using an infratuberositary approach [18]. After performing the osteotomy, the TDO was simulated by closing the osteotomy in increments of 6, 8, 10 and 12 mm while rotating the distal tibial fragment around the HA. The mMPTA was measured before and after each simulated closing distance of the baseline TDO. It was measured using the mechanical axis of the tibia and proximal articular surface of the tibia in the coronal plane according to [17]. Additionally, the change in the PTS was measured for every closing distance, and the change in the PTS per millimetre was calculated. Next, the HA and associated TDO plane were internally and externally rotated in increments of 5°, 10°, 15°, 20° and 30° around the tibial mechanical axis. For each degree of rotation, the TDO was simulated with the aforementioned closing increments, and the mMPTA was measured for each degree of rotation and closing distance (Figure 2). Furthermore, the rotational differences between the baseline TDO and the following three TDO orientations were measured: (1) perpendicular to the tibial tuberosity, (2) perpendicular to a lateral knee radiograph with a perfect overlay of the posterior femoral condyles and (3) perpendicular to a lateral knee radiograph with a perfect overlay of the posterior tibial condyles (Figure 3). Because high tibial osteotomies achieve an mMPTA correction accuracy of approximately 2° [21], a postoperative change of ≥ 2° in the mMPTA was defined as a relevant change. All TDO simulations and mMPTA measurements were performed by two independent readers (MK, GN) to determine inter‐rater reliability. The intraclass correlation coefficients (ICC) between the two readers showed almost perfect agreement for measuring the preoperative mMPTA [0.999 (95% CI: 0.996–1.000)] and for measuring the mMPTA after the simulated osteotomies [0.997 (95% CI: 0.996–0.997)]. Mean values were used for further analyses.

### Statistical analysis

Sociodemographic and clinical characteristics of the patients were summarised using descriptive statistics. Continuous variables are reported as mean ± standard deviation (SD). Normality of distribution was tested using the Shapiro‐Wilk test. Accordingly, a two‐tailed paired t‐test or Wilcoxon test was applied to assess the differences between pre‐ and postoperative (post‐simulation) parameters. Post hoc power analysis revealed a power of 100% to detect significant differences in mMPTA at an *α*‐value of 0.05, given the sample size. The analyses were performed using SPSS for Windows (version 29.0, SPSS Inc., Chicago, Illinois, USA). Statistical significance was set at *p* < 0.05.

## RESULTS

Forty TDOs were simulated using the baseline TDO plane, and the concomitant mMPTA was measured. Mean change of PTS per millimetre was 0.9 ± 0.0° (range: 0.9–0.9). Additionally, 400 TDOs using internally and externally rotated HA were simulated, and the concomitant mMPTA was measured. The mean mMPTA values are presented in Table 1. The absolute differences between the pre‐ and postoperative mMPTA measurements are summarised in Table 2. The TDO perpendicular to the tibial tuberosity was, on average, 5.1 ± 3.5° (range: −1.3 to 10.1) externally rotated compared with the baseline TDO. The TDO perpendicular to the lateral knee radiograph with a perfect overlay of the posterior femoral condyles was, on average, 14.5 ± 6.7° (range: 2.2–24.9) internally rotated, and the TDO perpendicular to the lateral knee radiograph with a perfect overlay of the posterior tibial condyles was, on average, 12.8 ± 6.4° (range: 5.1–25.9) internally rotated compared with the baseline TDO.

**Table 1:** Mean mechanical medial proximal tibial angle (mMPTA) measurements after tibial deflexion osteotomy with varying degrees of hinge axes (HA) rotations and different closing distances of the osteotomy.

Closing distance	Osteotomy angulation	Degree of hinge axis rotation					
		0°	5°	10°	15°	20°	30°
6 mm	ER	88.4° ± 1.2° *p* = n.s.	88.7° ± 1.2° *p* = 0.004	89.1° ± 1.2° *p* = 0.002	89.5° ± 1.2° *p* = 0.002	89.9° ± 1.2° *p* = 0.002	90.6° ± 1.2° *p* = 0.002
	IR		88.0° ± 1.1° *p* = 0.006	87.4° ± 1.1° *p* = 0.002	87.0° ± 1.1° *p* = 0.002	86.7° ± 1.1° *p* = 0.002	86.0° ± 1.1° *p* = 0.002
8 mm	ER	88.4° ± 1.2° *p* = n.s.	88.9° ± 1.2° *p* = 0.004	89.4° ± 1.2° *p* = 0.002	89.9° ± 1.2° *p* = 0.002	90.4° ± 1.2° *p* = 0.002	91.3° ± 1.2° *p* = 0.002
	IR		87.8° ± 1.2° *p* = 0.002	87.2° ± 1.1° *p* = 0.002	86.7° ± 1.1° *p* = 0.002	86.2° ± 1.1° *p* = 0.002	85.3° ± 1.1° *p* = 0.002
10 mm	ER	88.4° ± 1.2° *p* = n.s.	89.0° ± 1.2° *p* = 0.004	89.6° ± 1.2° *p* = 0.002	90.3° ± 1.2° *p* = 0.002	90.9° ± 1.2° *p* = 0.002	92.1° ± 1.2° *p* = 0.002
	IR		87.7° ± 1.2° *p* = 0.002	86.9° ± 1.1° *p* = 0.002	86.3° ± 1.1° *p* = 0.002	85.7° ± 1.1° *p* = 0.002	84.5° ± 1.2° *p* = 0.002
12 mm	ER	88.4° ± 1.2° *p* = n.s.	89.1° ± 1.2° *p* = 0.004	89.9° ± 1.2° *p* = 0.002	90.6° ± 1.2° *p* = 0.002	91.4° ± 1.2° *p* = 0.002	92.8° ± 1.3° *p* = 0.002
	IR		87.6° ± 1.2° *p* = 0.002	86.7° ± 1.1° *p* = 0.002	85.9° ± 1.1° *p* = 0.002	85.2° ± 1.2° *p* = 0.002	83.8° ± 1.2° *p* = 0.002

*Note*: *p*‐Values refer to the preoperative mMPTA.

Abbreviations: ER, external rotation; IR, internal rotation.

**Table 2:** Absolute difference between pre‐ and postoperative mechanical medial proximal tibial angle (mMPTA) measurements after tibial deflexion osteotomy with different hinge axes (HA) rotations and closing distances of the osteotomy.

Closing distance	Degree of internal‐ and external rotation of the hinge axis				
	± 5°	± 10°	± 15°	± 20°	± 30°
6 mm	0.4° ± 0.1°	0.8° ± 0.3°	1.2° ± 0.3°	1.6° ± 0.3°	**2.3°** ± **0.3°**
8 mm	0.5 ° ± 0.1°	1.1° ± 0.3°	1.6° ± 0.3°	**2.1°** ± **0.3°**	**3.0°** ± **0.4°**
10 mm	0.6° ± 0.1°	1.4° ± 0.3°	**2.0°** ± **0.3°**	**2.6°** ± **0.3°**	**3.8°** ± **0.4°**
12 mm	0.8° ± 0.1°	1.6° ± 0.3°	**2.3°** ± **0.3°**	**3.1°** ± **0.4°**	**4.5°** ± **0.5°**

*Note*: Values ≥ 2° are shown in bold.

## DISCUSSION

The most important finding of this study is that internal and external rotation of the HA in TDO may cause significant and relevant changes in postoperative mMPTA. The mMPTA changed significantly with an HA rotation of only 5°. However, postoperative changes of the mMPTA exceeded the relevant threshold of ≥ 2° when the HA was internally or externally rotated by 30° in small osteotomy closing distances (i.e. 6 mm), and by 15° in larger closing distances (i.e., ≥10 mm). Thus, the study hypothesis was confirmed.

PTS is recognised as a main risk factor for graft failure in patients with recurrent ACL ruptures [13]. To address this issue, TDO has become an important treatment option in this patient population. Several studies have reported that TDO results in low graft rupture rates and improves patient reported outcomes [1, 4, 14, 23, 24]. Although a PTS of ≥12° has traditionally been regarded as the threshold for recommending TDO in the context of ACL revision surgery, recent literature suggests that this cut‐off can be lowered to 10.1° [5, 12]. Various surgical techniques for TDO have been described in literature, including supratuberositary, tuberosity‐reflecting transtuberositary, and infratuberositary osteotomies [4, 23, 24]. Although the supratuberositary approach is technically demanding and the tuberosity‐reflecting transtuberositary technique requires an additional osteotomy, the infratuberositary approach is technically more feasible, preserves patellar height, and is associated with low complication rates [15, 19, 20, 22]. These factors may explain the recent popularity of the infratuberositary approach. However, unintended postoperative changes in the mMPTA have been observed even after this approach [2, 16]. Helm et al. [8] showed in a computer simulation study that performing TDO with different anterior starting points (i.e., centred over the tibial tuberosity or middle of the tibial head) resulted in different changes in the postoperative mMPTA. However, they analysed the mediolateral translation of the osteotomy entry point, and although the starting point probably altered the HA orientation, the resulting rotational changes were not accounted. The change in mMPTA can be explained by the different HA orientations [25]. This principle was applied to combined corrections of the PTS and coronal leg axis [9, 26]. However, when TDO is performed for isolated PTS correction, preservation of the postoperative mMPTA is essential. This study showed that TDO with the HA perpendicular to the sagittal plane and osteotomy plane orientation perpendicular to the coronal plane in the standardised long‐leg‐radiograph position resulted in no significant change in the postoperative mMPTA. Achieving such an HA orientation intraoperatively can be challenging, and the use of navigations aids such as patient‐specific instruments (PSI) could be helpful [26]. Most surgeons still perform TDO using a freehand technique under fluoroscopic guidance. The tibial tuberosity or a lateral knee radiography can be used to determine intraoperative orientation during osteotomy navigation [7, 18]. Our results suggest that to preserve preoperative mMPTA, the osteotomy plane should be internally rotated by 5.1° from the tibial tuberosity or externally rotated 14.5° and 12.8° from the perpendicular plane of the lateral knee radiograph with a perfect overlay of the posterior femoral and tibial condyles, respectively. These values may serve as a general orientation for a conventional freehand TDO intended for the isolated correction of the PTS.

This study has a few limitations, and its findings should be interpreted accordingly. First, a computer simulation approach was used to calculate the changes in the mMPTA, and possible soft‐tissue constraints and other in vivo biomechanical factors could not be considered in such an approach. Cadaver experiments can provide a more comprehensive analysis than computer simulations; however, they are labour‐intensive and costly. Therefore, a computer simulation is an established and cost‐effective alternative. Second, the study had a small sample size. However, although the number of study patients was limited owing to the strict inclusion and exclusion criteria, the number is likely sufficient to support a generalisable conclusion regarding changes in mMPTA associated with different HA orientations in infratuberositary TDO. Lastly, the described HA orientation for preserving the preoperative mMPTA is challenging to perform without navigation aids. The mean internal and external rotations reported for the tibial tuberosity orientation and lateral knee radiograph orientation with an overlay of the posterior femoral and tibial condyles relative to the baseline TDO should be regarded as approximate values. The limited number of included bone models does not provide a definitive recommendation for HA orientation in TDO to preserve preoperative mMPTA. Therefore, a larger patient cohort is required to confirm these recommendations.

## CONCLUSION

A TDO oriented perpendicular to the leg's coronal plane preserves the preoperative mMPTA and therefore avoids unintended coronal correction. The mMPTA changed significantly with a rotation of the HA of only 5° and exceeded a postoperative change of ≥ 2° with 15° of HA rotation.

## AUTHOR CONTRIBUTIONS

All authors have made substantial contributions to all of the following: (1) the conception and design of the study, or acquisition of data, or analysis and interpretation of data, (2) drafting the article or revising it critically for important intellectual content, (3) final approval of the version to be submitted.

## CONFLICT OF INTEREST STATEMENT

The authors declare no conflicts of interest.

## ETHICS STATEMENT

The local ethical committee approved this retrospective study (Zurich Cantonal Ethics Commission, BASEC‐Nr. 2023‐00389). Informed consent was obtained from all study participants.

## Data Availability

Data available on request from the authors.
